# Procalcitonin as a prognostic marker for sepsis based on SEPSIS‐3

**DOI:** 10.1002/jcla.22996

**Published:** 2019-08-16

**Authors:** Dong Wook Jekarl, Seungok Lee, Myungshin Kim, Yonggoo Kim, Seon Hee Woo, Woon Jeong Lee

**Affiliations:** ^1^ Department of Laboratory Medicine The Catholic University of Korea, Seoul St. Mary’s Hospital Seoul Korea; ^2^ Laboratory for Development and Evaluation Center The Catholic University of Korea Seoul Korea; ^3^ Department of Laboratory Medicine The Catholic University of Korea, Incheon St. Mary’s Hospital Incheon Korea; ^4^ Department of Emergency Medicine The Catholic University of Korea, Incheon St. Mary’s Hospital Incheon Korea

**Keywords:** bacteremia, diagnosis, procalcitonin, prognosis, sepsis

## Abstract

**Background:**

The revised definition of sepsis is life‐threatening organ dysfunction caused by a dysregulated host response to infection (SEPSIS‐3). The objective of this study was to evaluate procalcitonin (PCT) for the diagnosis and prognosis of sepsis using SEPSIS‐3.

**Methods:**

We enrolled 248 patients, who were admitted to the emergency department with suspected bacterial infection from June 2016 to February 2017. Definite bacterial infection was defined by proven culture results, and probable bacterial infection was based on diagnostic modalities other than culture. The sequential organ failure assessment (SOFA) score of 2 points or more from the baseline was diagnosed as sepsis. PCT was measured by the AFIAS‐6 immunoassay system (Boditech Med Inc.) using whole blood. White blood cell (WBC), C‐reactive protein (CRP), and erythrocyte sedimentation rate (ERS) were evaluated.

**Results:**

The final diagnosis was sepsis in 185 patients with infection of respiratory and genitourinary tract constituted 84.6%. The area under the receiver operating characteristic curve (AUROC) with 95% confidence interval (CI) was as follows: PCT, 0.682 (0.589‐0.765); CRP, 0.583 (0.487‐0.673); ESR, 0.540 (0.515‐0.699); and WBC, 0.611 (0.455‐0.633), respectively. In multivariate analysis, age, SOFA, and PCT (log scale) predicted non‐survivors with an odds ratio with 95% confidence interval of 1.055 (1.008‐1.105), 1.303 (1.142‐1.486), and 2.004 (1.240‐3.238), respectively. Among sepsis group, initial PCT was increased in non‐survivor (23.2 ng/dL) compared to survivor group (8.1 ng/dL) with statistical significance (*P* = .005).

**Conclusions:**

PCT could support and predict the unfavorable prognosis of sepsis based on SEPSIS‐3, whereas diagnostic potential of PCT requires further evaluations.

## 1 INTRODUCTION

The original concept of sepsis, which was defined as a systemic inflammatory response syndrome (SIRS) with documented microbial infection, has been used for more than two decades.^1^ This definition was revised in part due to an improved understanding of the pathobiology of sepsis.^2^, ^3^, ^4^ Sepsis is now regarded as early activation of both pro‐ and anti‐inflammatory responses with involvement of non‐immunologic systems including cardiovascular, endocrine, and coagulation.^5^ In addition, the low diagnostic capability of SIRS led to a revision of the definition of sepsis, though SIRS criteria might still be useful for the identification of infection.^3^, ^4^

The revised definition of sepsis proposed in 2016 (SEPSIS‐3) is life‐threatening organ dysfunction caused by a dysregulated host response to infection.^5^ In brief, former conditions of sepsis and severe sepsis are now regarded as bacterial infection and sepsis, respectively. The revised definition emphasizes organ dysfunction, which can be calculated by sequential organ failure assessment (SOFA) score. The SOFA score‐based definition of sepsis predicted mortality higher than that of SIRS‐based definition. As infection can lead to organ failure, patients with infection should be carefully followed up.^5^ For ease of application in clinical environments, the laboratory data included in SOFA score were bilirubin, creatinine, and platelet count.

There have been continuous attempts to diagnosis of SIRS, sepsis, and severe sepsis using biomarkers, especially procalcitonin (PCT), C‐reactive protein (CRP), white blood cell (WBC), erythrocyte sedimentation rate (ESR), and various interleukins.^6^, ^7^, ^8^ PCT and biomarkers are debated for the usefulness and clinical application, but the previous literatures revealed that PCT could support the clinical diagnosis and treatment of patients. Among these biomarkers, PCT and CRP were included in the diagnostic criteria of inflammatory variables in Surviving Sepsis Campaign 2013.^9^ However, in the Surviving Sepsis Campaign 2016, PCT was revised to be a recommended biomarker for sepsis prognosis but not for diagnosis.^10^ Both diagnosis and prognosis are important in sepsis, and several biomarkers including PCT, sTREM‐1, presepsin, and cytokines have been studied for predicting prognosis under former definition of sepsis.^7^, ^11^, ^12^, ^13^, ^14^ In addition, PCT is related to antimicrobial stewardship, a treatment that encompasses initiation and tapering of antimicrobial treatment.^15^, ^16^

The biomarkers evaluated for previously defined severe sepsis might not reflect the performance in revised sepsis due to differences in the details of definitions. In addition, diagnosis of sepsis was revised suing SOFA score, which requires three clinical variables and three laboratory variables, leading to a score range from zero to 24. We hypothesized that the PCT, CRP, WBC, and ESR might result in capabilities to diagnose sepsis and reflect prognosis. Diagnostic capability of biomarkers could be evaluated by the area under the receiver operating characteristic curve (AUROC) value, and prognostic capability of biomarkers could be evaluated by univariate and multivariate analysis. Therefore, in this study, we evaluated accessible biomarkers including PCT, CRP, ESR, and WBC in the clinical setting for their utility in the diagnosis and prognosis of revised sepsis.

## 2 PATIENTS AND METHODS

### 2.1 Patients in the study cohort

This was a single‐center study performed at a tertiary teaching hospital, and the study protocol was approved by the institutional review board of Incheon St. Mary's Hospital. Individual consent was not required by the institutional review board because the data were obtained during the course of diagnosis and treatment. Patients (≥18 years of age) admitted to the emergency department of Incheon St. Mary's Hospital who were diagnosed with suspected bacterial infection by an emergency department physician were enrolled from June 2016 to February 2017. A total of 248 patients were enrolled from 124 male and 124 female, respectively.

After the patients were admitted to Emergency Department, routine venous blood sampling was performed before administration of therapeutics. Suspected infection was defined as clinical assessment of signs and symptoms, laboratory and radiologic results, concomitant administration of oral or parenteral antibiotics, and sampling of body fluid cultures including blood, urine, cerebrospinal fluid, and peritoneal fluid.^5^, ^17^

Patients were excluded from the study if they had evidence of an immunocompromised state (eg, malignancy) or of a viral infection including respiratory virus or hepatitis virus. Hepatitis A viral infection was excluded if anti‐HAV antibody was negative. Acute hepatitis B viral infection was excluded if HBsAg, anti‐HBc antibody, and HBeAg were negative. Chronic Hepatitis B viral infection was excluded if duration of HBsAg positivity was <6 months. Hepatitis C viral infection was excluded if anti‐HCV was negative. Respiratory virus infection was excluded if the interpretation of radiologic study was negative or 14 kinds of multiplex virus PCR (influenza A, influenza B, parainfluenza 1,2,3, respiratory syncytial virus A, B, adenovirus, metapneumovirus, rhinovirus, three kinds of coronavirus and bocavirus) were negative.^18^, ^19^ Demographic data and baseline characteristics of patients were collected at the time of admission.

### 2.2 Diagnosis of definite and probable bacterial infection

Bacterial infection was diagnosed based on clinical manifestation, laboratory results, recovery of pathogens, and radiologic studies.^5^, ^17^, ^20^ The diagnosis of definite bacterial infection was defined as positive microbial culture results of body fluids including blood, sputum, urine, pleural fluid, peritoneal fluid, and cerebrospinal fluid among patients with suspected bacterial infection. The diagnosis of probable bacterial infection was based on medical examinations as follows: microbiological tests excluding culture of body fluids or without recovery of pathogens by culture; immunochromatographic methods; real‐time or conventional polymerase chain reaction (PCR); radiologic analyses, including X‐ray, ultrasonography, and computed tomography; and serology.^5^, ^17^ Patients with suspected bacterial infection were followed up by investigators via hospital medical records until discharged from the hospital.

### 2.3 Diagnosis of sepsis

Among enrolled patients, data on the following component of SOFA score were collected and graded: PaO_2_/FiO_2_ (mm Hg), platelet count, bilirubin, Glasgow Coma Scale (GCS) score, creatinine or renal output level, and mean arterial pressure. The mean arterial pressure was calculated as follows: diastolic blood pressure – 1/3 × (systolic blood pressure – diastolic blood pressure). Enrolled patients with suspected bacterial infection and SOFA score of 2 points or more from the baseline were diagnosed with sepsis.^5^

### 2.4 Laboratory examination

Routine microbiological examination included more than two pair of blood cultures. Samples were cultured using two sets of aerobic and anaerobic bottles (BACTEC plus). BACTEC automated blood culture system (BD Biosciences) was used for incubation for 5 days.^21^ Culture and analysis of various body fluids (urine, sputum, broncho‐alveolar lavage fluid, cerebrospinal fluid, abscess, and closed wound) were performed. Blood samples for WBC counts, ESR, PCT, CRP, and blood chemistry were drawn immediately after presentation to the emergency department and were analyzed in a central laboratory within 2 hours. Hematologic parameters including WBC were measured by Sysmex XN2000 (Sysmex). High‐sensitivity C‐reactive protein (CRP) was measured by a latex agglutination method using a Beckman Coulter AU5400 Automated Biochemistry Analyzer (Beckman Coulter). ESR was determined by a quantitative capillary photometry method using Test‐1 (Alifax). PCT was measured with whole blood using an AFIAS PCT immunoassay (Boditech Med Inc) which quantitatively measures PCT. Fluorescent immunoassay was used and the diagnostic precision was analyzed for 20 working days, and duplicated runs were measured two times within the working day (2 × 2 × 20 protocol) and coefficient of variation (CV) was calculated. The CV of low level (1.089 ng/mL) and high level material (11.69 ng/mL) was as follows: repeatability, 6.51%, 5.65%; between‐run, 9.73%, 8.12%; between‐day, 8.08%, 7.27%; within‐laboratory, 14.2%, 12.3%, respectively (Table S2).^22^ The limit of blank and limit of detection provided by the manufacturer were 0.044 and 0.066 ng/mL. The claimed analytical measurement range was from 0.1 ng/mL to 100 ng/mL. Cut‐off value provided by the manufacturer was 0.5 ng/mL.

### 2.5 Statistical analysis

Comparisons of 63 non‐sepsis patients and 185 sepsis patients were performed using Student's *t* test for continuous variables, or the chi‐square test or Fisher's exact test for categorical variables. Diagnostic performance of PCT, CRP, ESR, and WBC counts was analyzed using AUROC, which were compared using a non‐parametric method. The cut‐off value was selected as the maximum value of sensitivity and specificity. Sensitivity, specificity, positive and negative predictive values, and accuracy were calculated with 95% confidence interval. Prediction of non‐survivors was performed by univariate analysis, and variables with statistical significance were analyzed in multivariate analysis. Univariate analysis was performed with a single variable by the logistic regression analysis, and the multivariate analysis was performed using the variables from the univariate analysis that was statistically significant.^19^, ^23^ Comparison between survivor and non‐survivor was performed for sepsis group with Mann‐Whitney *U* test. Statistical analyses and figures were generated using Medcalc software version 18.0 (Medcalc).

## 3 RESULTS

### 3.1 Comparison of non‐sepsis and sepsis groups

The cohort consisted of 248 patients diagnosed with suspected bacterial infection who were initially admitted to the emergency department. Of the 248 patients, 63 were classified as the non‐sepsis group and 185 as sepsis group. Table 1 shows a comparison of demographic and baseline data between the non‐sepsis group and the sepsis group. The mean age, PCT, and WBC of patients in the sepsis group were significantly higher than that of patients in the non‐sepsis group. The identified bacteria or other microorganisms are listed in detail in Table S1. Among the identified microbes, *Escherichia coli* (21.8%) was the most common pathogen, followed by *Klebsiella* species (13.8%) and *Staphylococcus aureus* (11.6%).

**Table 1:** Clinical characteristics and baseline demographics of patients

Characteristics	Total	Non‐sepsis	Sepsis	*P*
	n = 248	n = 63	n = 185	
Demographics				
Male/Female	124/124	29 /34	95 /90	NS
Age, y	70.1 ± 14.9	65.8 ± 14.7	71.5 ± 26.2	.009
Tested markers				
PCT (ng/mL)	8.5 ± 22.2	3.2 ± 11.7	10.2 ± 24.5	<.001
CRP (mg/L)	91.9 ± 66.9	78.5 ± 66.3	96.5 ± 66.5	NS
ESR (mm/hr)	48.6 ± 25.6	45.8 ± 25.1	49.6 ± 25.8	NS
WBC (×10^9^/L)	12.8 ± 7.5	10.8 ± 5.8	13.4 ± 7.9	.012
SOFA factors				
Respiratory factors				
PaO_2_ (mm Hg)	78.8 ± 25.1	91.2 ± 15.8	74.6 ± 26.2	<.001
FiO_2_ (mm Hg)	0.31 ± 0.15	0.22 ± 0.07	0.33 ± 0.17	<.001
PaO_2_/FiO_2_	307.4 ± 140.1	416.2 ± 100.2	270.2 ± 132.4	<.001
Platelets (×10^3^/uL)	231 ± 114	242 ± 92	227 ± 120	NS
Bilirubin (mg/dL)	0.99 ± 1.78	0.76 ± 0.35	1.1 ± 2.1	NS
MAP (mm Hg)	85.9 ± 24.2	89.9 ± 15.7	84.6 ± 26.3	.028
GCS score	13.4 ± 3.1	14.9 ± 0.45	12.9 ± 3.4	<.001
Creatinine (mg/dL)	1.8 ± 2.3	0.81 ± 0.31	2.19 ± 2.58	<.001
Suspected bacterial infection				NS
Definite bacterial infection	135	36	99	
Probable bacterial infection	113	27	86	
Final diagnosis				
Respiratory tract	116	26	90	NS
Genitourinary tract	94	27	67	NS
Gastrointestinal tract	3	2	1	NS
Hepato‐biliary tract	12	4	8	NS
Others	23	4	19	NS
Prognosis				.02
Survivor	220	61	159	
Non‐survivor	28	2	26	

Note

The continuous variables are listed as mean ± standard deviation.

Abbreviations: CRP, C‐reactive protein; ESR, erythrocyte sedimentation rate; FiO_2_, fraction of inspired oxygen; GCS, Glasgow Coma Scale; MAP, mean arterial pressure; NS, non‐specific; PaO_2_, partial pressure of oxygen; PCT, procalcitonin; WBC, white blood cell.

### 3.2 Diagnosis of sepsis among patients with suspected bacterial infection

**Figure 1:**
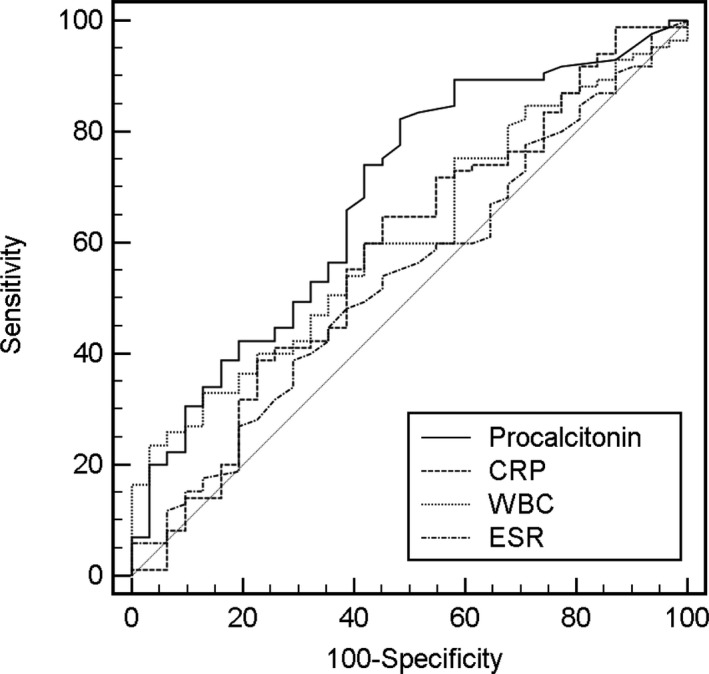
C‐reactive protein (CRP), erythrocyte sedimentation rate (ESR), curves. Procalcitonin (PCT), Receiver operating characteristic (ROC), and white blood cell (WBC) count are plotted

The ROC curves for PCT, CRP, ESR level, and WBC count for diagnosis of sepsis are shown in Figure 1. Table 2 lists the AUROC, cut‐off value, sensitivity, specificity, and positive and negative predictive values. Pairwise comparison was performed as follows: PCT vs ESR; PCT vs CRP; PCT vs WBC; ESR vs CRP; ESR vs WBC; CRP vs WBC, respectively. Comparison of AUROCs between biomarkers revealed that none of the biomarkers showed statistical significance.

**Table 2:** Diagnostic potential of tested biomarkers

Biomarkers	PCT (ng/mL)	CRP (mg/L)	ESR (mm/hr)	WBC (×109/L)
ROC (95% CI)	0.682 (0.589‐0.765)	0.583 (0.487‐0.673)	0.540 (0.515‐0.699)	0.611 (0.445‐0.633)
Cut‐off	0.18	51.16	48	13.66
Sensitivity (95% CI)	84.5 (78.4‐89.5)	66.5 (59.1‐73.3)	56.2 (46.2‐65.9)	41.0 (32.9‐47.4)
Specificity (95% CI)	46.6 (30.0‐55.9)	50.8 (37.9‐63.6)	55.3 (38.3‐71.4)	77.8 (65.5‐87.3)
PPV (95% CI)	61.2 (48.8‐73.7)	57.4 (44.8‐70.1)	55.7 (43.0‐68.3)	64.8 (52.6‐77.1)
NPV (95% CI)	75.1 (65.4‐84.6)	60.2 (49.4‐71.1)	55.8 (44.7‐66.8)	56.8 (45.8‐67.8)

Abbreviations: CI, confidence intervals; CRP, C‐reactive protein; ESR, erythrocyte sedimentation rate; NPV, negative predictive value; PCT, procalcitonin; PPV, positive predictive value; ROC, receiver operating characteristic curve; WBC, white blood cell.

### 3.3 Prediction of non‐survivors

Among the age, sex, and biomarkers, age, PCT, WBC, and SOFA score revealed statistical significance in univariate analysis (Table 3A). With these variables, multivariate analysis was performed. Among them, age, SOFA score, and PCT (log scale) predicted non‐survivors with statistical significance in multivariate analysis (Table 3B). The age, SOFA, and PCT (log scale) predicted non‐survivors with an odds ratio and 95% confidence interval of 1.055 (1.008‐1.105), 1.303 (1.142‐1.486), and 2.004 (1.240‐3.238), respectively.

**Table 3:** Prediction of non‐survivors among patients with sepsis. A, Univariate analysis of variable. B, multivariate analysis

A			
Non‐survivor	Univariate		
	*P* value	Odd ratio	95% CI
Sex	NS		
Age	.012	1.048	1.011‐1.088
PCT (log scale)	<.001	2.251	1.441‐3.517
CRP	NS		
ESR	NS		
WBC	.025	1.052	1.006‐1.099
SOFA score	<.001	1.365	1.187‐1.548

B			
Non‐survivor	Multivariate		
	*P* value	Odd ratio	95% CI
Sex			
Age	.022	1.055	1.008‐1.105
PCT (log scale)	.005	2.004	1.240‐3.238
CRP			
ESR			
WBC	NS		
SOFA score	<.001	1.303	1.142‐1.486

Abbreviations: CRP, C‐reactive protein; ESR, erythrocyte sedimentation rate; PCT, procalcitonin; SOFA, sequential organ failure assessment WBC, white blood cell.

### 3.4 Comparison of survivor and non‐survivor among sepsis

Comparison of survivor (n = 159) and non‐survivor (n = 26) was performed among sepsis group (Table 4). PCT was higher in non‐survivor group compared to survivor group. Among tested markers, only PCT revealed statistical significance (*P* = .005). Hemoglobin was lower in non‐survivor group. Most of SOFA score components revealed statistical significance except for the platelets and bilirubin.

**Table 4:** Comparison of survivor and non‐survivor among sepsis group

Characteristics	Survivor	Non‐survivor	*P*
	n = 159	n = 26	
Demographics			
Male/Female	80/79	15/11	NS
Age, y	70.7 ± 14.9	76.7 ± 9.3	NS
Tested Markers			
PCT (ng/mL)	8.1 ± 19.5	23.2 ± 43.2	.005
CRP (mg/L)	94.0 ± 66.9	111.7 ± 63.6	NS
ESR (mm/hr)	50.7 ± 25.9	42.9 ± 24.7	NS
WBC (×10^9^/L)	13.0 ± 6.7	16.1 ± 12.7	NS
Laboratory data			
Hg (g/dL)	11.6 ± 2.3	10.5 ± 2.5	.027
SOFA components			
SOFA score	3.54 ± 2.81	4.65 ± 3.12	<.001
Respiratory factors			
PaO_2_ (mm Hg)	74.5 ± 22.6	76.0 ± 43.2	NS
FiO_2_ (mm Hg)	0.31 ± 0.16	0.44 ± 0.21	<.001
PaO_2_/FiO_2_	281.4 ± 130.0	202.1 ± 128.6	.002
Platelets (×10^3^/uL)	230 ± 122.5	202.1 ± 128.6	NS
Bilirubin (mg/dL)	1.11 ± 2.20	0.84 ± 0.42	NS
MAP	86.3 ± 26.2	74.1 ± 25.4	.008
GCS score	13.0 ± 3.5	12.3 ± 3.1	.026
creatinine (mg/dL)	1.97 ± 2.46	3.57 ± 2.94	<.001
Suspected bacterial infection			NS
Definite bacterial infection	88 (47.5)	11 (5.9)	
Probable bacterial infection	71 (38.3)	15 (8.1)	

Note

The continuous variables are listed as mean ± standard deviation.

Abbreviations: CRP, C‐reactive protein; ESR, erythrocyte sedimentation rate; FiO_2_, fraction of inspired oxygen; GCS, Glasgow Coma Scale; Hg, hemoglobin; MAP, mean arterial pressure; NS, non‐specific; PaO_2_, partial pressure of oxygen; PCT, procalcitonin; WBC, white blood cell.

## 4 DISCUSSION

The revised definition of sepsis might require accumulated data for validation and overcome controversies. The original concept of severe sepsis was defined as SIRS patients with documented bacterial infection together with organ dysfunction.^1^ For predicting mortality, severe sepsis revealed higher sensitivity and specificity of 92.0% and 84.0%, respectively, compared with those of SOFA and qSOFA.^16^, ^24^ SIRS criteria might be useful for earlier signs of infection before development of organ dysfunction.^16^, ^24^

The diagnosis of sepsis depends on SOFA score that is the result of composite score from PaO_2_/FiO_2_ (mm Hg), platelet count, bilirubin, Glasgow Coma Scale (GCS) score, creatinine or renal output level, and mean arterial pressure with documented infection. Although there is a quick SOFA score for the detection of sepsis outside of intensive care unit, diagnosis of sepsis was hindered by heterogeneous nature of sepsis including wide range of sign and symptoms, host immune response, immune‐compromised state of patient, and various microbes.

Therefore, using biomarkers has undeniable merit for diagnosis and prognosis and additional information should be provided that is unavailable from established clinical tests.^25^ Various biomarkers related to pathobiology have been studied including PCT, presepsin, and cytokines.^6^, ^26^, ^27^ Although there is controversy over the use of PCT, few biomarkers have outperformed PCT for diagnosis and prognosis of bacterial infection or sepsis.^6^, ^28^, ^29^, ^30^ Previous studies based on the former definition of sepsis revealed that the AUROC of PCT was approximately 0.7‐0.8.^6^, ^7^, ^8^

Under the revised definition of sepsis, our data on the diagnostic performance of PCT yielded an AUROC of 0.682, which was slightly lower than expected compared with the literature. One of the possible reasons for the low AUROC could be a control group. As control group used in this study also had bacterial infection without sepsis, this group also showed increased PCT level of 3.2 ng/mL and 36 out of 63 cases had definite bacterial infection. These 36 cases of definite bacterial infection proven by culture might have been classified as a sepsis based on the previous sepsis definition. If healthy control groups or systemic inflammatory response syndrome group were recruited as a control group, the AUROC might be been increased. As PCT is expected to reflect a bacterial infection with or without organ dysfunction, further studies are required for the diagnostic performance of PCT under revised sepsis definition. Other biomarker that specifically reflects organ dysfunction might be required for accurate sepsis diagnosis.

Unlike diagnostic performance, the prognostic performance of PCT was better than expected and the odd ratio of PCT (log scale) was 2.004 (95 CI, 1.240‐3.238), which was higher than SOFA score and demographic parameters. The probability of unfavorable prognosis increased associated with higher PCT concentration. These results were in line with previous data that the PCT predicted prognosis of sepsis patients.^30^ Further studies are required for prognostic utility of PCT based on new definition.

There was no statistical difference between the non‐survivor and survivor group for CRP, ESR, and WBC. Lower hemoglobin level in non‐survivor group implies that oxygenation or oxygen supply is associated with survival or underlying chronic disease might have affected the survival. Although age was higher in sepsis group, relatively small sample size might have resulted in statistical insignificance. Higher PCT concentration in non‐survivor group is related to unfavorable prognosis that was in line with multivariate model in this study.

The revised sepsis definition includes immune dysregulation, which requires to be measured. Some cytokines are suspected to be related to immune dysregulation, and the exact pathobiology must be identified. Cytokines or immune‐regulated molecules are complex, and network analysis might reveal pathobiology. Network analysis revealed that the sepsis network was small in size and path length was short,^31^ which might reflect immune dysregulation. PCT was one of the molecules that were the hub node among sepsis network,^31^ which implies that PCT is expected to play an important role among cytokine network in sepsis and interacted with other molecules.

The limitation of this study was that approximately 80% of patients were diagnosed with respiratory tract and genitourinary tract infection, which might differ from conditions in other hospital intensive care units or emergency departments. Age was increased in sepsis group, and age was one of the prognostic factors that predicted unfavorable prognosis. This was a single‐center study, and patient population might have affected the prevalence of sepsis group.

In conclusion, under the revised definition of sepsis, PCT could support prognosis of sepsis and predicted mortality compared with other parameters. Further studies are required to accumulate data on PCT using the revised sepsis definition for patient diagnosis and management.
