# Supplementary material for: Procalcitonin as a prognostic marker for sepsis based on SEPSIS‐3
Source: J Clin Lab Anal. 2019 Aug 16;33(9):e22996. doi: 10.1002/jcla.22996 (PMC6868407; doi:10.1002/jcla.22996)
Supplement: Supplementary file 1 [file JCLA-33-na-s001.docx]

**Supplementary Table 1.** Etiology of bacterial infection of patients. Some patients had more than onemicroorangism within a single specimen or in different clinical specimens.

| Etiology | Prevalence |
| --- | --- |
| Gram positive bacteria |  |
| *Staphylococcus aurues* | 16 |
| *Staphylococcus*spp | 3 |
| *Enterococcus* spp | 4 |
| *Streptococcus agalactiae* | 2 |
| *Streptococcus*spp | 3 |
| Gram negative bacteria |  |
| *Escherischia coli* | 53 |
| *Klebsiella*spp | 19 |
| *Pseudomonas*spp | 9 |
| *Acinetobacter*spp | 7 |
| Others |  |
| *Mycobacterium tuberculosis* | 7 |
| Non mycobacterium tuberculosis | 5 |
| *Clostridium*spp | 2 |
| *Candida*spp | 1 |
| *Proteus*spp | 1 |
| *Stenotrophomonas*spp | 1 |
| *Serratia*spp | 1 |
| *Enterobacter*spp | 1 |
| *Providentia*spp | 1 |
| *Morganella*spp | 1 |
| Total | 137 |
