# Supplementary material for: Procalcitonin as a prognostic marker for sepsis based on SEPSIS‐3
Source: J Clin Lab Anal. 2019 Aug 16;33(9):e22996. doi: 10.1002/jcla.22996 (PMC6868407; doi:10.1002/jcla.22996)
Supplement: Supplementary file 2 [file JCLA-33-na-s002.docx]

**Supplementary Table 2.** Precision analysis of the PCT.

|  | Mean | Repeatability | |  | Between-run | |  | Between-day | |  | Within-laboratory | |
| --- | --- | --- | --- | --- | --- | --- | --- | --- | --- | --- | --- | --- |
|  | (ng/mL) | SD (95% CI) | CV (%) |  | SD (95% CI) | CV (%) |  | SD (95% CI) | CV (%) |  | SD (95% CI) | CV (%) |
| Level 1 | 1.089 | 0.071 (0.058 - 0.091) | 6.51 |  | 0.106 (0.087 - 0.135) | 9.73 |  | 0.088 (0.1445 - 0.225) | 8.08 |  | 0.155 (0.131 - 0.188) | 14.2 |
| Level 2 | 11.69 | 0.66 (0.54 - 0.84) | 5.65 |  | 0.95 (0.780 - 1.216) | 8.12 |  | 0.85 (0.705 -1.099) | 7.27 |  | 1.44 (1.298 - 1.862) | 12.3 |

Abbreviation: CV, coefficient of variation; SD, standard deviation
